# Multicenter registry study of cerebral venous thrombosis in china (RETAIN‐CH): Rationale and design

**DOI:** 10.1002/brb3.3353

**Published:** 2024-04-15

**Authors:** Hetao Bian, Xia Wang, Lan Liu, Feng Yan, Shan Lu, Wen Hui, Chen Zhou, Jiangang Duan, Min Li, Jian Chen, Ran Meng, Lei Cao, Longde Wang, Xunming Ji

**Affiliations:** ^1^ Beijing Institute for Brain Disorders Capital Medical University Beijing China; ^2^ The George Institute for Global Health University of New South Wales Sydney Australia; ^3^ School of Statistics University of Minnesota at Twin Cities Minneapolis Minnesota USA; ^4^ Department of Neurosurgery Xuanwu Hospital of Capital Medical University Beijing China; ^5^ Department of Neurology and Psychiatry Beijing Shijitan Hospital Capital Medical University Beijing China; ^6^ Department of Science and Technology West China Hospital of Sichuan University Chengdu Sichuan China; ^7^ Department of Emergency Xuanwu Hospital of Capital Medical University Beijing China; ^8^ Department of Neurology Xuanwu Hospital of Capital Medical University Beijing China; ^9^ The General Office of Stroke Prevention Project Committee National Health Commission of the People's Republic of China Beijing China

**Keywords:** cerebral venous thrombosis, cohort study, endovascular treatment, protocol, safety

## Abstract

**Background and rationale:**

Cerebral venous thrombosis (CVT) is a rare cerebrovascular disorder that mainly affects young and middle‐aged adults. Epidemiological data on the incidence, risk factors, diagnosis, treatment, and prognosis of CVT are lacking in China. In addition, there is a lack of evidence from large, multicenter, real‐world studies on the efficacy and safety of endovascular.

**Aim:**

To understand the incidence, diagnosis and treatment status of CVT in China and to estimate the effectiveness and safety of endovascular treatment in the real‐world.

**Methods:**

A multicenter, retrospective observational cohort study will be conducted on CVT patient records from 104 hospitals, between January 1, 2018 and June 30, 2022, identified using a 2‐stage cluster sampling design based on per capita gross domestic product. Each enrolled participant is required to complete a further follow‐up, which includes the current situation and the assessment at 3 and 12 months after discharge.

**Study outcomes:**

The outcomes of this study will include the current status of the incidence, pathogenesis, etiology, clinical symptoms, diagnosis, and treatment of CVT in China, as well as the effectiveness and safety of endovascular treatment in the real‐world.

**Discussion:**

Results from this study will provide evidence on the incidence, specific risk factors, symptomatic and imaging features, and clinical outcomes of CVT in China as well as indicate whether endovascular treatment is superior to medical management alone for patients with acute CVT in the real‐world.

**Trial registration:**

http://www.clinicaltrials.gov.

**Identifier:**

NCT05448248

## Abstract 2

This is a protocal about our study. This protocal introduces the design, method and content of our research.

## 1 INTRODUCTION AND RATIONALE

Cerebral venous thrombosis (CVT) is a rare cerebrovascular disorder that mainly affects young and middle‐aged adults, especially women (Otite et al., 2020). Early estimates of the incidence of CVT ranged from 0.1 to 0.2 cases per 100,000 population (Silvis et al., 2017). However, data from population‐based studies in recent years indicate that the current incidence among adults is much higher than this estimate (Devasagayam et al., 2016). This may be due to changes in risk factors and improvements in imaging techniques. The common causes of CVT are diseases and risk factors that lead to a hypercoagulable state. These include genetic prothrombotic conditions, acquired prothrombotic states, infections, inflammatory disease, hematological disease, drugs, mechanical causes, and trauma (Piazza, 2012). Recent studies suggest that the prevalence of risk factors varies widely in different countries (Salottolo et al., 2017; Silvis et al., 2017). The current incidence and risk factors of CVT in China are unclear.

Systemic anticoagulation is currently the main treatment and standard of care for CVT (Ropper & Klein, 2021). Endovascular treatment for CVT has shown favorable results in case series and single‐center studies (Jedi et al., 2022; Salottolo et al., 2017; Siddiqui et al., 2015). An international multicenter randomized trial comparing endovascular treatment with heparin therapy was terminated early. Patients are selected based on a predicted poor prognosis, and the effectiveness of endovascular therapy is uncertain (Coutinho et al., 2020).

Therefore, we designed a large epidemiological multicenter retrospective observational cohort study to evaluate the current status of the incidence, diagnosis and treatment of CVT in China, as well as the efficacy and safety of endovascular treatment in the real‐world.

## 2 METHODS

### 2.1 Study design

The RETAIN‐CH is a multicenter, retrospective observational cohort study using patient clinical data. The registration number is NCT 05448248. It will examine more than 3000 hospitalizations for CVT from a nationally representative network of Chinese hospitals between January 2018 and June 2022. This study has been designed according to the STROBE criteria.

### 2.2 Sampling design

The hospitals were chosen to reflect the morbidity, diagnosis, treatment, and prognosis status of CVT in mainland China. CVT is a rare and easily misdiagnosed disease, which is more common in provincial and prefecture‐level hospitals and rarely seen in county‐level hospitals, according to our preliminary investigation. Therefore, only provincial and prefecture‐level hospitals were selected in this study. As hospital volumes and clinical capacities differ between provincial capitals and prefectural‐level cities in mainland China, we separately identified hospitals in two strata: provincial capital cities and prefectural level cities.

**FIGURE 1:**
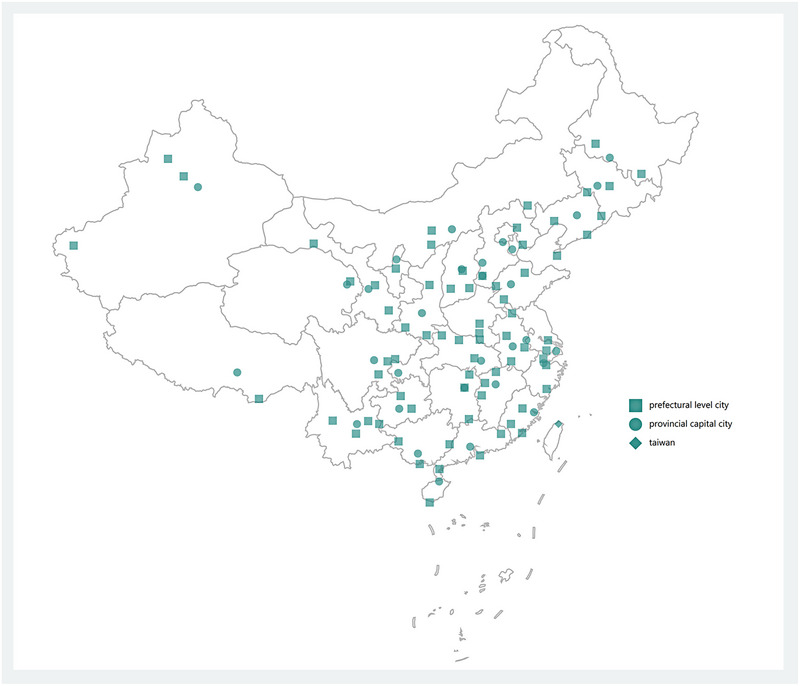
Map of study sites participated in China mainland for the RETAIN‐CH study geographic distribution of participating hospitals in the RETAIN‐CH study. Of 104 sampled hospitals, 31 were in provincial capital cities, and 73 were in prefectural level cities.

A stratified two‐stage cluster sampling design was used to determine the cases to be included in this study. In the first stage, we selected the capital city and three prefecture‐level cities in each province. All the prefecture‐level cities in each province were stratified as developed, developing and undeveloped according to the gross domestic product per capita level in 2021. Then, one prefecture‐level city was randomly selected from the three prefecture‐level cities, respectively. In this way, each province will draw four cities, including one provincial capital and three prefecture‐level cities. We considered a capital city if it is a direct‐controlled municipality (Beijing, Shanghai, Tianjin, Chongqing). For provinces with few prefecture‐level cities or city‐level hospitals (Hainan, Tibet, Qinghai, Ningxia), only one prefecture‐level city was randomly selected. In the second stage, we employed a random sampling method to select one tertiary hospital from each of the sampled cities. Following the previously mentioned stratified sampling approach, 104 hospitals were selected to participate in the RETAIN‐CH study (Figure 1).

### 2.3 Patient population

Inclusion criteria: (1) Patients with CVT at each institution were initially identified using ICD‐9 (325.0, 437.6, and 671.5) or ICD‐10 codes (I63.6, I67.6, O22.5X, O87.3, and G08) (Otite et al., 2020; Yaghi et al., 2022). This was followed by the review of medical records and imaging studies to confirm the diagnosis of CVT. (2) Patients providing informed consent.

Exclusion criteria: (1) Patients with second and subsequent admissions of CVT are will excluded, (2) CVT not confirmed by imaging, and (3) denial of informed consent.

### 2.4 Study outcomes

#### The following variables were collected through medical record review to understand the current status of diagnosis and treatment of CVT in China

1. Demographic factors: age, gender, and ethnicity.
2. Clinical variables: days from onset, clinical symptoms (headache, dizziness, nausea, vomiting, seizures, visual disturbances, motor deficits, sensory changes, mental status disorder, aphasia, altered mental status, coma, tinnitus, tinnitus cerebri, hearing disorder, discomfort in the neck, xerophthalmia, etc.), and modified Rankin scale (mRS).
3. CVT risk factors: hypertension, diabetes, hyperlipemia, hyperuricemia, hyperhomocysteinemia, history of stroke, history of headache, history of lower limb venous thrombosis, history of pulmonary embolism, history of heart failure, tumor history, history of autoimmune disease, history of infection, history of hematological diseases, history of head trauma within 3 months of CVT diagnosis, surgical history, history of dehydration, history of nephrotic syndrome, genetic thrombophilia, vaccination history, history of structural heart disease, history of oral diet drugs, smoking history, drinking history, history of oral contraceptives, history of abortion, history of artificial assisted reproduction, pregnancy, delivery within 8 weeks of CVT diagnosis, and body mass index.
4. Imaging variables: brain imaging type [computed tomography, computed tomographic venography, magnetic resonance imaging, magnetic resonance venography, or digital subtraction angiography], brain imaging findings (venous infarct, cerebral edema, brain hemorrhage, or subarachnoid hemorrhage), and CVT location (superior sagittal sinus, transverse sinus, straight sinus, sigmoid sinus, cavernous sinus, deep cerebral veins, venae cortical, jugular vein, or other veins).
5. Laboratory variables: platelet count, hemoglobin, red blood cell count, international normalized ratio, prothrombin time, thrombin time, activated partial thromboplastin time, fibrinogen, D‐dimer, and homocysteine.
6. In‐hospital treatments: drug treatment (unfractionated heparin, low molecular weight heparin, warfarin, dabigatran, rivaroxaban, apixaban, batroxobin, aspirin, clopidogrel, hormone therapy, anti‐infective therapy, dehydration treatment, anti‐epileptic therapy, and analgesic therapy), endovascular treatment (arterial thrombolytic therapy, thrombolytic therapy of venous sinuses, mechanical thrombectomy, and stent‐support angioplasty), and neurosurgical treatment (craniotomy, ventricle shunt surgery, and minimally invasive removal of intracranial hematoma).
7. Outcomes at discharge: mRS; mortality; discharge medications (unfractionated heparin, low molecular heparin, warfarin, dabigatran, rivaroxaban, apixaban, aspirin, clopidogrel, anti‐epileptic drugs, antidepressants, and folic acid); the length of hospital stay; hospitalization expenses; and the proportion of hospitalized patients with CVT and stroke.

#### The following variables were collected during follow‐up to further clarify the efficacy and safety of endovascular treatment

Each enrolled participant was required to complete a further follow‐up, which included the current situation and the assessment at 3 and 12 months after discharge. Follow‐up assessments must include mRS, epilepsy, and palindromia at 3 and 12 months after discharge. Follow‐up evaluation of the current status must include mRS, patient health questionnaire 9‐item (PHQ‐9), headache impact test 6‐items (HIT‐6), epilepsy, palindromia, currently on oral medication.

The primary outcome to be tested is the proportion of patients achieving mRS score (0−1) in the current situation.

Secondary outcomes: (1) the proportion of mRS score (0−1) at 3 months after discharge, (2) the proportion of mRS score (0−1) at 12 months after discharge, (3) the proportion of mRS score (0−2) in the current situation, (4) the proportion of mRS score (0−2) at 3 months after discharge, (5) the proportion of mRS score (0−2) at 12 months after discharge, (6) the proportion of mRS score (0−2) at 12 months after discharge, (7) the PHQ‐9 score in the current situation, and (8) the HIT‐6 score in the current situation. Safety variables were mortality at discharge, at 3 and 12 months after discharge, and at present.

### 2.5 Data collection

All data collection will be conducted electronically using a bespoke database on Big Data Observatory platform for Stroke in China. It is encrypted and accessed by individual username and password. Data administrators or data entry clerk will undertake the data collection. Each branch has one data administrator and multiple data entry clerks. Each variable in the database will be collected in a standardized format.

All study staff from participating hospitals were trained using a standardized protocol and formally certified before study commencement. The training included how to identify all hospitalizations for CVT from their respective local hospital databases, how to complete the electronic case report form, and how to use the follow‐up assessment scale. Each participating hospital has a professional data manager to verify and monitor the quality and integrity of the data, and ensure compliance with standardized research protocols. Once a potential data error is identified, the data manager will trace and review the relevant records to resolve the problem. During the study period, the quality and completeness of study in each participating hospital would be checked regularly by the project quality management team.

### 2.6 Data management

The source data for RETAIN‐CH will be collected from participants’ medical databases and follow‐up visits. After we sampled cases at each hospital, we assigned each case a unique study ID. All data are treated as protected health information and securely stored in an encrypted and password‐protected database at the coordinating center. In the process of data entry, if data are found missing, the data registration system displays a warning to remind the data entry clerk to confirm and complete the data. For some data that need to be filled in specific values, such as time, weight, height, and laboratory test results, if there are unusual values during data entry, the data cannot be saved, and the data entry clerk will be reminded to check the data. Details of the data manager's role, such as checking for missing data, unusual values (range checks) will be specified in the RETAIN‐CH data management plan. Detailed initiatives for monitoring the data will be presented in the RETAIN‐CH monitoring plan.

### 2.7 Sample size estimates

No target recruitment number has been set for the RETAIN‐CH registry. We anticipate the inclusion of approximately 3000–3500 subjects based on the preliminary study.

### 2.8 Statistical analysis

Statistical analyses will be performed using SAS software (version 9.3) and the R programming language. The *t*‐test will be used for two independent samples of continuous variables, and analysis of variance will be used for three or more sets of continuous data. Comparisons of frequency, percentage, and ratio will be made using the *χ* ^2^ test or Fisher's exact test. To study the relationship between variables, linear regression will be used for continuous dependent variables, and logistic regression or Cox regression will be used for classified dependent variables. In addition to help account for the nonrandomized treatment, propensity‐score methods will be used to reduce the confounding effects. Statistical significance is indicated by *p* < .05.

## 3 DISCUSSION

The RETAIN‐CH is a national multicenter retrospective enrollment study. The participating clinical subcenters were randomly selected in 31 provinces and municipalities according to regional, economic and medical levels, for better representation. A total of 104 clinical subcenters were randomly selected to participate in the study. This study will determine the incidence, clinical symptoms, etiology, diagnosis, treatment, and prognosis of CVT patients in China in the last 5 years, in the real‐world. This study will also indicate whether endovascular treatment is superior to medical management alone in patients with acute CVT in the real‐world.

CVT is a type of stroke that mainly affects young and middle‐aged adults, especially women (Silvis et al., 2017). Many countries have reported the proportion of CVT in stroke patients, the proportion of incidence in different age groups, and the proportion of incidence by gender (Coutinho et al., 2009; Otite et al., 2020; Rezoagli et al., 2021; Ruuskanen et al., 2021), but there is no relevant representative data in China. The RETAIN‐CH study will report the proportion of CVT in stroke patients, as well as demographic data such as age and gender in China.

The symptoms presented by patients with CVT are highly variable. Acute or subacute headache is the most common initial symptom of CVT and is reported by 70%−90% of patients (Ropper & Klein, 2021). Specific risk factors for CVT such as thrombophilia, oral contraceptive use, pregnancy and puerperium, cancer, inflammatory conditions, and head trauma have been described early (Silvis et al., 2017), but the composition of risk factors may be changing worldwide. The RETAIN‐CH study will clarify the distribution of risk factors for CVT in China in recent years.

The treatment of CVT has been a research hotspot in recent years. Presently, the treatment of CVT typically involves anticoagulation therapy, endovascular treatment, and supportive care. Endovascular therapy may be an option in certain cases, particularly when there are severe acute symptoms and life‐threatening conditions. The efficacy and safety of endovascular treatment and the optimal duration of anticoagulant therapy are some of the urgent clinical issues to be addressed (Bucke et al., 2022; Silvis et al., 2017). The RETAIN‐CH study will elucidate the current status of different treatment strategies in China and analyze the effectiveness and safety of endovascular treatment in the real‐world. Moreover, the long‐term outcome of the patients will be further evaluated by follow‐up.

In summary, the RETAIN‐CH study is intended to elucidate the demographic data, proportion of stroke admissions, clinical symptoms, risk factors, diagnosis, treatment, burden of disease, and prognosis of CVT patients in China. This study will provide new evidence for the endovascular treatment of CVT in the real‐world.

In the last few years, major progress has been made in the epidemiology, diagnosis, and treatment of CVT. Although CVT is currently considered to have good prognosis, there are also many problems that need to be further explored and solved. There is a greater need for international cooperation in the future to further understand the genetics, related disorders, and treatments of this multifaceted disease.

## AUTHOR CONTRIBUTIONS

All the authors were responsible for the conception and design of the study. Hetao Bian, Xia Wang, Lan Liu, Lei Cao, Wen Hui, Xunming Ji *designed the registry*; Hetao Bian, Feng Yan, and Shan Lu *drafted the manuscript*; Jian Chen, Chen Zhou, Jiangang Duan, Min Li, Ran Meng, Longde Wang, and Xunming Ji *revised and commented the manuscript*. All authors read and approved the final version of the manuscript.

## CONFLICT OF INTEREST STATEMENT

The authors declare that they have no conflicts of interest.

### PEER REVIEW

The peer review history for this article is available at https://publons.com/publon/10.1002/brb3.3353.

## Data Availability

The data that support the findings of this study are available on request from the corresponding author.
